# Nrf2 regulates glucose uptake and metabolism in neurons and astrocytes

**DOI:** 10.1016/j.redox.2023.102672

**Published:** 2023-03-14

**Authors:** Noemí Esteras, Thomas S. Blacker, Evgeny A. Zherebtsov, Olga A. Stelmashuk, Ying Zhang, W. Christian Wigley, Michael R. Duchen, Albena T. Dinkova-Kostova, Andrey Y. Abramov

**Affiliations:** a Department of Clinical and Movement Neurosciences, UCL Queen Square Institute of Neurology, Queen Square, London, WC1N 3BG, UK; b Research Department of Cell & Developmental Biology, University College London, Gower Street, London, WC1E 6BT, UK; c Optoelectronics and Measurement Techniques, University of Oulu, Oulu, Finland; d Laboratory of Cell Physiology and Pathology, Orel State University, Orel, Russia; e Jacqui Wood Cancer, Division of Cellular Medicine, School of Medicine, University of Dundee, Dundee, DD1 9SY, Scotland, UK; f Reata Pharmaceuticals, 2801 Gateway Dr, Suite 150, Irving, TX, 75063, USA; g Departments of Medicine and Pharmacology and Molecular Sciences, Johns Hopkins University School of Medicine, Baltimore, MD, 21205, USA

**Keywords:** Nrf2, Glucose metabolism, Mitochondria, NADH, NADPH, Brain, Neurons, Astrocytes

## Abstract

The transcription factor Nrf2 and its repressor Keap1 mediate cell stress adaptation by inducing expression of genes regulating cellular detoxification, antioxidant defence and energy metabolism. Energy production and antioxidant defence employ NADH and NADPH respectively as essential metabolic cofactors; both are generated in distinct pathways of glucose metabolism, and both pathways are enhanced by Nrf2 activation. Here, we examined the role of Nrf2 on glucose distribution and the interrelation between NADH production in energy metabolism and NADPH homeostasis using glio-neuronal cultures isolated from wild-type, Nrf2-knockout and Keap1-knockdown mice. Employing advanced microscopy imaging of single live cells, including multiphoton fluorescence lifetime imaging microscopy (FLIM) to discriminate between NADH and NADPH, we found that Nrf2 activation increases glucose uptake into neurons and astrocytes. Glucose consumption is prioritized in brain cells for mitochondrial NADH and energy production, with a smaller contribution to NADPH synthesis in the pentose phosphate pathway for redox reactions. As Nrf2 is suppressed during neuronal development, this strategy leaves neurons reliant on astrocytic Nrf2 to maintain redox balance and energy homeostasis.

## Highlights

• Nrf2 increases glucose uptake in neurons and astrocytes.
• Nrf2 activation enhances both NADH and NADPH cytoplasmic levels in basal conditions.
• Nrf2 favours energy production in astrocytes when glucose availability is reduced.
• Glucose is prioritized in brain for mitochondrial NADH and energy production.

## 1 Introduction

The nuclear factor erythroid-derived 2 (NF-E2)-related factor 2 (Nrf2) is encoded by the gene *NFE2L2* and belongs to the family of basic leucine zipper (bZIP) transcription factors. Its activation is cytoprotective by inducing the expression of genes that encode detoxifying enzymes and that enhance cellular antioxidant defence. Detoxifying and antioxidant enzymes such as thioredoxin, peroxiredoxin, NAD(P)H:quinone oxidoreductase 1 (NQO1) and glutathione reductases require the cofactor NADPH. Considering the upregulation of these enzymes by Nrf2 [1], the rate of turnover of NADPH in cells with high Nrf2 levels also increases [2]. NADPH is the major reducing resource to scavenge pro-oxidants and to regenerate glutathione and thioredoxin; and it is also required for anabolic pathways including lipid and nucleic acid synthesis. Importantly, NADPH is also used in an opposite way to its antioxidant role when supporting redox signalling – by providing substrate for NADPH oxidases, leading to the production of superoxide anion and hydrogen peroxide, and for NO synthesis. Thus, constitutive activation of Nrf2 by knock-down of its repressor Keap1 (Keap1-KD) was shown to upregulate NADPH oxidase and contribute to NADPH consumption in glio-neuronal cultures [3]. NADPH in the brain is mainly produced in the pentose phosphate pathway (PPP), with a smaller role for cytosolic and mitochondrial isocitrate dehydrogenase and malic enzyme 1 [[4], [5], [6]]. It has been shown that Nrf2 activation increased PPP activity in fibroblasts, liver cells and astroglia [2,7,8]. Importantly, in response to Nrf2 activation, glucose was shown to be preferentially utilised in the PPP for NADPH production in fibroblasts [7], and in anabolic pathways supporting cell proliferation in cancer cells [9]. Interestingly, in cells with different metabolic profiles such as brain cells, Nrf2 activation increased the PPP flux in astroglial cells, but reduced it in neurons, pointing at differential roles of Nrf2 in this context, which needs to be further explored [8].

Nrf2 is not only an antioxidant and detoxification activator, but also plays an important role in mitochondrial and intermediary metabolism as part of its cytoprotective activity [10]. Thus, Nrf2 activation by Keap1 knock-down, enhances mitochondrial fatty acid oxidation [11]. Importantly, Nrf2 activation increases ATP production in neurons and astrocytes through the stimulation of substrate delivery (mostly NADH and FADH_2_) in mitochondria [12,13]. Indeed, increased energy supply following activation of Nrf2 in neurons has been shown to be protective in several neurodegenerative disease models and in epilepsy [[14], [15], [16], [17]]. Brain energy metabolism is a complex system involving neuron-glia metabolic interactions. ATP production in the brain mostly involves glucose utilization by glycolysis, culminating in the oxidation of its final product pyruvate by the mitochondrial Krebs cycle. This results in the generation of cofactors such as NADH and FADH_2_ that are used to produce ATP by oxidative phosphorylation. Thus, stimulation of energy metabolism in brain cells by Nrf2 activation should also significantly increase glucose consumption for these processes.

It is not clear how enhanced glucose metabolism through the PPP to promote antioxidant defence correlates in the same cells with increased glucose consumption by glycolysis for ATP production. Moreover, it remains to be elucidated how these processes can be equilibrated in cells with very different metabolic requirements such as neurons and astrocytes, which additionally establish profound metabolic and functional interactions. To study this, we generated primary mixed cultures of neurons and astrocytes isolated from Nrf2-knockout, Keap1-KD and WT mice. To understand how Nrf2 activity influenced glucose distribution between the pathways, we monitored the essential metabolic cofactors involved in each process: NADH generated by glycolysis and Krebs cycle for energy production, and NADPH generated by PPP for use in antioxidant defence, after exposing the cells to different conditions. Given the complexity and heterogeneity of the cultures, with mixed populations of cellular types, the most commonly used biochemical approaches are not suitable for this purpose. We therefore assessed the levels of both cofactors (mitochondrial and enzyme-bound cytoplasmic NADH and NADPH) by using advanced multiphoton fluorescence lifetime imaging microscopy (FLIM) of their combined, spectrally identical autofluorescence (labelled NAD(P)H) in order to separate their signals in single live cells [18]. We found that the rate of glucose uptake was significantly higher in Keap1-KD cells compared to Nrf2-KO and WT cells. Activation of Nrf2 by Keap1-KD increased enzyme-bound cytoplasmic NADPH and NADH and particularly mitochondrial NADH in both neurons and astrocytes. We found that when glucose availability was limiting, astrocytic Nrf2 prioritized the metabolism of glucose for energy production. Importantly, inhibition of glycolysis did not lead to the redistribution of glucose for use in the PPP; while conversely, following inhibition of the PPP, Nrf2 activation led to higher levels of NADH. These findings suggest that Nrf2 activation prioritizes mitochondrial NADH and energy production, with a smaller contribution to promote NADPH synthesis in the PPP for redox reactions in brain cells.

## 2 Results

### 2.1 Keap1 knockdown activates glucose uptake in neurons and astrocytes

**Fig. 1:**
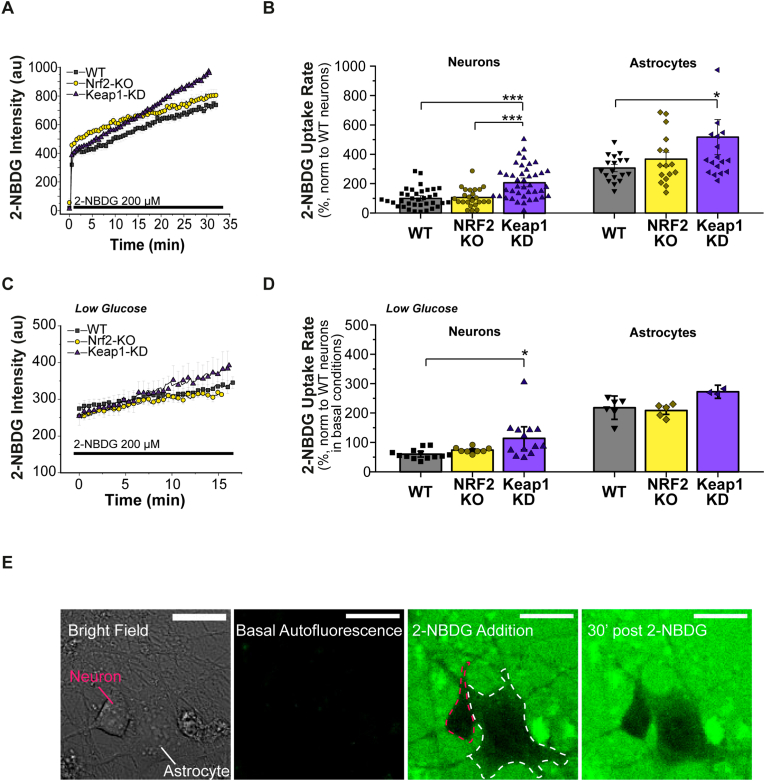
***Keap1 KD increases glucose uptake in neurons and astrocytes*** Glucose uptake rate measured as the rate of increase in 2-NBDG fluorescence intensity inside cells over time. Representative traces (A) and quantification (B) of the glucose uptake rate, normalized to WT neurons. WT n = 37; Nrf2-KO n = 24; Keap1-KD n = 41 neurons and WT = 18; Nrf2-KO n = 16, Keap1-KD n = 19 astrocytes. Representative traces (C) and quantification (D) of the glucose uptake rate after incubation in low glucose (1 mM) HBSS for 12 h. WT n = 13; Nrf2-KO n = 8, Keap1-KD n = 13 neurons and WT n = 6, Nrf2-KO n = 5, Keap1-KD n = 3 astrocytes. (E) Representative images of an experiment to measure 2-NBDG uptake rate in a neuron and an astrocyte, cell types outlined as indicated. 2-NBDG addition to the media leads to an increase in extracellular green fluorescence. Uptake of the fluorescent glucose leads to an increase in the fluorescent signal inside the cells. Scale bar: 20 μm. One-way ANOVA with Bonferroni post-hoc analysis for each group *p < 0.05, ***p < 0.0001. (For interpretation of the references to colour in this figure legend, the reader is referred to the Web version of this article.)

**Supplementary Fig. 1:**
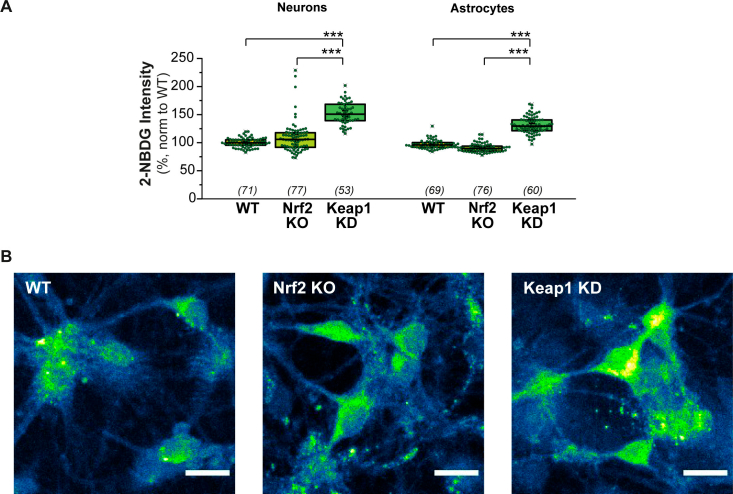
Nrf2 activation increases glucose uptake in neurons and astrocytes (A) Glucose uptake measured as the intensity of the fluorescent glucose analogue 2-NBDG inside cells after 30 min incubation with the dye and subsequent wash of the media. Number of cells analysed indicated in brackets. Non-parametric Kruskal-Wallis ANOVA, ***p < 0.0001. (B) Representative images of 2-NBDG intensity. Scale bar: 20 μm.

To investigate glucose uptake by primary neurons and astrocytes we used the fluorescent glucose analogue 2-NBDG as explained in Methods (Fig. 1, Supplementary Fig. 1). The rate of 2-NBDG uptake in Nrf2-deficient neurons and astrocytes was similar to that in WT (Fig. 1 A, B, Supplementary Fig. 1). However, in Keap1-KD neurons (208 ± 112%, n = 41) and astrocytes (517 ± 353%, n = 17) the rate of glucose uptake was ∼2-fold greater than in the WT cells (p < 0.05 in neurons 100 arb u./min ±65%, n = 37, p = 10^−7^ and in astrocytes 306 ± 87%, n = 18, p = 0.02) (Fig. 1 A, B, Supplementary Fig. 1). A similar difference between genotypes was also observed in cell cultures incubated in medium with low glucose for 12 h, but the rate of 2-NBDG uptake was slower (WT 59 ± 16%, n = 13; Keap1-KD 113 ± 68%, n = 13, p = 0.01; Fig. 1C, D). Thus, the rate of glucose uptake was increased in Keap1-KD neurons and astrocytes compared to WT cells.

### 2.2 Mitochondrial NADH levels are modulated by Nrf2 activity

**Fig. 2:**
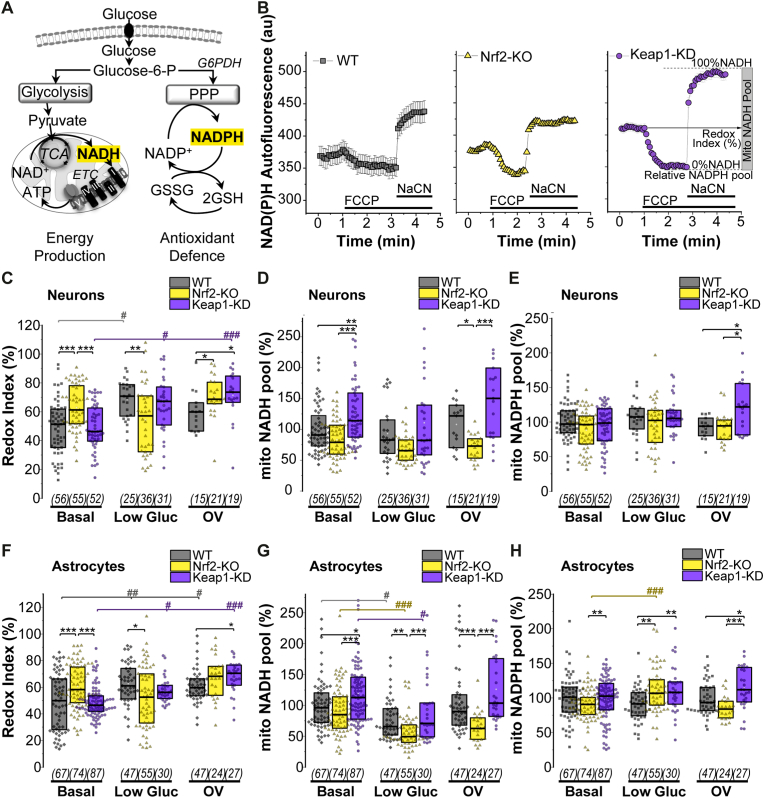
***Nrf2 affects mitochondrial NADH levels in neurons and astrocytes and NADPH levels in astrocytes*** (A) Scheme showing main pathways of cellular glucose metabolism: glycolysis resulting in NADH production in the mitochondria and pentose phosphate pathway (PPP), resulting in NADPH production. (B) Representative traces showing mitochondrial NADH homeostasis, as measured by its autofluorescence, in astrocytes. Uncoupler FCCP maximizes respiration, leading to consumption of all mitochondrial NADH. Remaining fluorescence at this stage is attributed to NADPH. Subsequent application of NaCN inhibits respiration leading to mitochondrial NADH accumulation. Redox index is the relative NADH level in basal conditions of the total mitochondrial NADH pool. (C-H) Quantification of: redox index (C,F), mitochondrial NADH pool (D,G), and relative NADPH pool (E,H) as explained in (B) in WT, Nrf2KO and Keap1 KD individual neurons and astrocytes. Basal (25 mM glucose), low glucose (12 h incubation in 1 mM glucose) and orthovanadate (Basal plus OV 30 μM for 12 h). Mitochondrial NADH and NADPH pools are expressed as a % of basal WT. Number of cells analysed is shown in brackets. Non-parametric Kruskal-Wallis ANOVA with post-hoc Dunn's test for each group, *p < 0.05, **p < 0.01, ***p < 0.0001.

Metabolism of glucose by glycolysis and oxidation of its final product, pyruvate, in the mitochondrial tricarboxylic acid (TCA) cycle leads to the production of NADH, which is used by the electron transport chain (ETC) to generate ATP by oxidative phosphorylation (Fig. 2A). To understand the role of Nrf2 in modulating glucose utilization for energy production, we explored mitochondrial NADH homeostasis under different scenarios of glucose availability: basal conditions, diminished extracellular glucose concentration, and increased glucose utilization in the presence of the phosphatase inhibitor orthovanadate, known to act as an insulin-mimetic [19,20].

To assess the mitochondrial pool of NADH in the different conditions, we measured NADH autofluorescence in the mitochondria of individual cells as described in Methods and previously published [21] (Fig. 2B). Addition of the uncoupler FCCP maximizes respiration, leading to the oxidation of the mitochondrial NADH pool to NAD^+^, which is not fluorescent. This point of minimum fluorescence is therefore taken as 0% mitochondrial NADH pool. Subsequent addition of the ETC inhibitor, NaCN, blocks respiration and prevents oxidation of NADH, bringing NADH levels to maximal values (taken as 100%, see Fig. 2B). This approach helps us to estimate NADH-related mitochondrial respiration in intact cells (as a balance between reduction and oxidation of NADH – referred to as the ‘redox index’) and the NADH pool in mitochondria.

In agreement with our previous observations [12], Nrf2 deficiency induced a decrease in respiration in both neurons (p = 10^−4^) (Fig. 2C) and astrocytes (p = 10^−4^), as suggested by the higher redox index (Fig. 2F). Interestingly, incubation of cells with limited extracellular glucose (1 mM, 12 h) also decreased respiration in WT and Keap1-KD neurons (p = 10^−4^, p = 0.01) and astrocytes (p = 0.009; p = 0.04), compared to basal conditions. Treatment of the cells with the phosphatase inhibitor orthovanadate (OV, 30 μM, 12 h), known to increase glucose utilization, also elevated the redox index in WT astrocytes (p = 0.03) and Keap-1KD neurons (p = 10^−4^) and astrocytes (p = 10^−5^) (Fig. 2C, F) relative to basal, suggesting that this treatment also diminished the respiration rate.

The mitochondrial NADH pool in neurons and astrocytes was higher in Keap1-KD compared to WT (p = 0.006; p = 0.03) and Nrf2-KO cells (p = 10^−4^, p = 10^−4^) (Fig. 2 D, G). Importantly, limitation of the extracellular level of glucose to 1 mM decreased the mitochondrial NADH pool in all WT, Nrf2-KO and Keap1-KD-astrocytes, with a more pronounced effect in Nrf2-KO (WT, p = 0.01; Nrf2KO p = 10^−4^, Keap1-KD p = 0.03) (Fig. 2 D, G). Orthovanadate treatment on the other hand, did not affect the mitochondrial NADH pool in any of the genotypes compared to basal conditions. Thus, the level of NADH in mitochondria of neurons and astrocytes depends on the activity of Nrf2; and in astrocytes is also influenced by the availability of extracellular glucose.

The remaining NAD(P)H autofluorescence measured in mitochondria after consumption of all the mitochondrial NADH pool induced by FCCP can be taken as the mitochondrial NADPH pool (Fig. 2B). The basal level of mitochondrial NADPH was similar between WT, Nrf2-KO and Keap1-KD neurons, and elevated in Keap1-KD vs. Nrf2-KO astrocytes (p = 0.02) (Fig. 2 E, H). Interestingly, incubation with low glucose increased mitochondrial NADPH in Nrf2-KO astrocytes (Fig. 2 E, H). In WT and Keap1-KD the level of mitochondrial NADPH in cells was independent on the levels of glucose in the medium (Fig. 2 E, H), but, when incubated with OV, which enhances glucose utilization, it increased significantly in both Keap1-KD neurons and astrocytes compared to treated WT and Nrf2-KO cells. Thus, the level of mitochondrial NADPH in neurons and astrocytes is less dependent on the level of glucose in the medium compared to NADH.

### 2.3 FLIM measurements confirm that Nrf2 activation increases both enzyme-bound cytoplasmic NADH and NADPH levels in neurons and astrocytes

NADH and NADPH have identical spectral properties, and when measuring its autofluorescence intensity by live-cell imaging it is necessary to use specific inhibitors (such in Fig. 2) to try to understand the contribution of each. However, NADH and NADPH differ in their fluorescence lifetime when they are bound to enzymes [18]. We therefore next employed multiphoton FLIM (fluorescence lifetime imaging microscopy) to discriminate between enzyme-bound cytoplasmic NADH and NADPH (in addition to mitochondrial as shown in Fig. 2).

**Fig. 3:**
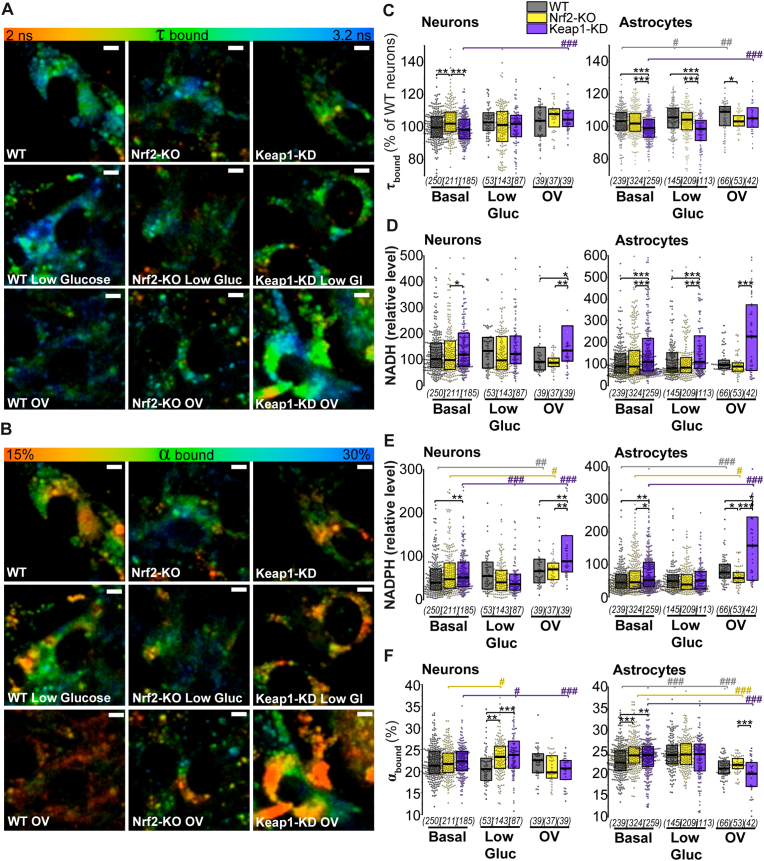
***Nrf2 activation enhances both cytoplasmic enzyme-bound NADH and NADPH levels in basal conditions, but favours energy production in astrocytes when glucose availability is reduced.*(A-B**) Colour-coded representative images of **(A)** NAD(P)H fluorescence lifetime (τ bound) and **(B)** percentage of enzyme-bound NAD(P)H (α bound) in basal conditions and after 12h incubation in low glucose (1 mM) media or orthovanadate (OV, 30 μM). **(C-F)** Quantification in individual neurons and astrocytes of **(C)** NAD(P)H fluorescence lifetime (τ bound); (D) relative cytoplasmic enzyme-bound NADH levels; (E) relative cytoplasmic enzyme-bound NADPH levels and (F) percentage of enzyme-bound NAD(P)H (α bound) in basal conditions and after 12h incubation in low glucose (1 mM) media or orthovanadate (OV, 30 μM). Number of cells analysed is shown in brackets. Scale bar: 5 μm. Non-parametric Kruskal-Wallis ANOVA with post-hoc Dunn's test for each group, *p < 0.05, **p < 0.01, ***p < 0.0001. (For interpretation of the references to colour in this figure legend, the reader is referred to the Web version of this article.)

Different parameters can be obtained from FLIM measurements. Among them, α-bound, which reflects the percentage of NAD(P)H bound to enzymes (Fig. 3 B, F), and τ-bound, which represents the fluorescence lifetime of NAD(P)H bound to enzymes (Fig. 3 A, C). Importantly, τ-bound reflects the ratio of enzyme-bound [NADPH] to [NADH] [18]. The relative contribution of NADH and NADPH to the total τ bound, can each be calculated on the basis of the different FLIM parameters as previously described [22,23]. Results show that in neurons, cytoplasmic relative levels of both enzyme-bound NADH (p = 0.03) and NAPDH (p = 0.001) are higher in Keap1-KD compared to Nrf2-KO and WT cells respectively (Fig. 3 D, E). Limited glucose availability abolishes the Keap1-KD advantage, and leads to similar enzyme-bound cytoplasmic NADH and NADPH levels between the genotypes (Fig. 3 D, E), as previously observed in the neuronal mitochondrial NADH pool (Fig. 2D). Keap1-KD astrocytes also show enhanced cytoplasmic levels of both enzyme-bound NADH (p = 10^−4^) and NADPH (p = 0.003) (Fig. 3 D, E) compared to WT, in agreement with observations in mitochondria (Fig. 2 G, H). When glucose availability in the media becomes limiting, Keap1-KD astrocytes maintain higher enzyme-bound cytoplasmic NADH levels than WT (p = 10^−4^) and Nrf2-KO (p = 10^−5^), while NAPDH levels are similar between the genotypes. This suggests that Nrf2 activation enhances both enzyme-bound cytoplasmic NADH and NADPH levels in basal conditions, but favours energy production over antioxidant defence when glucose availability is limited in astrocytes.

The presence of the insulin-mimetic OV, favoured NAPDH production. While OV treatment did not alter the basal differences between genotypes observed in enzyme-bound cytoplasmic NADH levels, NADPH levels were increased in WT (p = 0.001, p = 10^−8^), Nrf2-KO (p = 0.03, p = 0.05) and Keap1-KD (p = 10^−6^, p = 10^−6^) neurons and astrocytes, with Keap1-KD cells showing significantly higher levels than WT (p = 0.005, p = 0.02) and Nrf2-KO (p = 0.004, p = 10^−4^) (Fig. 3E). These results suggest that enhanced glucose utilization by OV favours flux into the PPP in preference to glycolysis, although other effects of OV in the glycolytic or PPP pathway cannot be fully excluded [24].

The percentage of enzyme-bound NAD(P)H can be estimated by α_bound._ It has been previously suggested that this parameter also correlates with the redox state of the cell, with higher alpha indicating a higher oxidation of the NADPH/NADH pool [18] (Fig. 3 B, F). Neurons presented similar percentage of enzyme-bound NAD(P)H between genotypes in basal conditions, while limited glucose availability increased its value in both Nrf2-KO and Keap1-KD relative to WT cells, suggesting an enhanced oxidation of the available pool regardless of Nrf2 activity (Nrf2-KO p = 0.01, Keap1-KD p = 0.04) (Fig. 3 B, F). In basal conditions, oxidation of the pool in astrocytes was higher in both Nrf2 deficient (p = 10^−6^) and in constitutively active cells (p = 0.002) as compared to WT (Fig. 3 B, F), while OV decreased the enzyme-bound percentage in all conditions (WT p = 10^−9^; Nrf2-KO p = 10^−7^, Keap1-KD p = 10^−8^) compared to basal (Fig. 3 F), suggesting a diminished oxidation of the available pool, (Fig. 3 F). The oxidation status of the NAD(P)H pool appeared to be independent of Nrf2 under any of these conditions suggesting that Nrf2 less likely regulates glucose sensing in neurons and astrocytes.

### 2.4 Inhibition of glycolysis does not lead to increased glucose utilization for the pentose-phosphate pathway

**Fig. 4:**
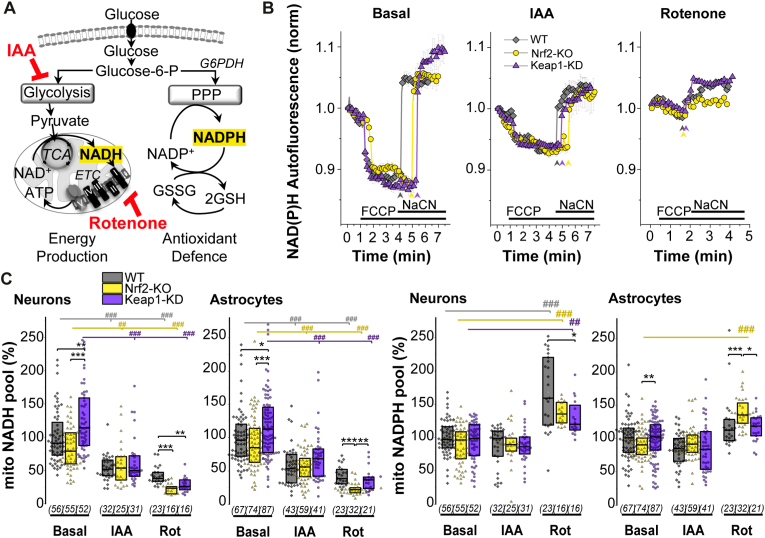
***Inhibition of glycolysis reduces mitochondrial NADH independently of Nrf2 status*****(A)** Scheme showing main pathways of cellular glucose metabolism and the role of iodoacetic acid (IAA) as an inhibitor of glycolysis (100 μM, 1 h, in the presence of 5 mM sodium pyruvate to maintain viability) and rotenone (rot) (5 μM, 20 min) as an inhibitor of complex I in the electron transport chain (ETC). **(B)** Representative traces of mitochondrial NADH homeostasis in neurons, in the absence/presence of the inhibitors, with arrows indicating the moment NaCN was added. **(C)** Quantification of the total mitochondrial NADH pool and **(D)** NADPH pool in mitochondria as measured by the remaining NAD(P)H autofluorescence after consumption of all mitochondrial NADH. Both are expressed as the % of WT basal. Number of cells analysed is shown in brackets. Non-parametric Kruskal-Wallis ANOVA with post-hoc Dunn's test for each group, *p < 0.05, **p < 0.01, ***p < 0.0001.

We next studied how the pharmacological manipulation of either the glycolytic or PPP pathways affected the metabolic profiles in each genotype, and how Nrf2 activation or deficiency influenced the outcome. We first focused on the glycolytic pathway and employed pharmacological modulators to block NADH production or consumption (Fig. 4A). Inhibition of glycolysis using Iodoacetic Acid (IAA, 20 μM for 1 h) led to a significant decrease in mitochondrial NADH in both neurons and astrocytes despite the support of NADH production in the TCA cycle induced with 5 mM sodium pyruvate to maintain viability (Fig. 4A–C). This effect was independent of the genotype (WT p = 10^−9^ p = 10^−8^: Nrf2-KO p = 0.01, p = 10^−6^, Keap1-KD p = 10^−7^, p = 10^−6^). Interestingly, inhibition of mitochondrial NADH consumption in complex I by rotenone (5 μM for 20 min) also significantly depleted the mitochondrial NADH pool in neurons and astrocytes of WT (p = 10^−12^, p = 10^−11^), Nrf2-KO (p = 10^−11^, p = 10^−24^) and Keap1-KD cells (p = 10^−12^, p = 10^−13^) (Fig. 4A–C). It should be noted that maximal decrease in mitochondrial NADH pool was observed in neurons and astrocytes with Nrf2-KO (Fig. 4C).

Rotenone-induced decrease in NADH led to a significant increase of the mitochondrial NADPH in neurons (measured as the residual autofluorescence after FCCP) independently of their genotype (WT p = 10^−5^, Nrf2-KO p = 10^−6^, Keap1-KD p = 0.003). These results, however, might be masked by the inability of mitochondria to consume all available NADH under FCCP treatment due to inhibition of complex I with rotenone. Interestingly, and mimicking exposure to low glucose (Fig. 2H), inhibition of mitochondrial complex I in Nrf2-KO astrocytes led to a higher mitochondrial NADPH compared to basal conditions (p = 10^−12^) and also to rotenone-treated WT (p = 10^−4^) and Keap-1 KD astrocytes (p = 0.01) (Fig. 4 D).

**Fig. 5:**
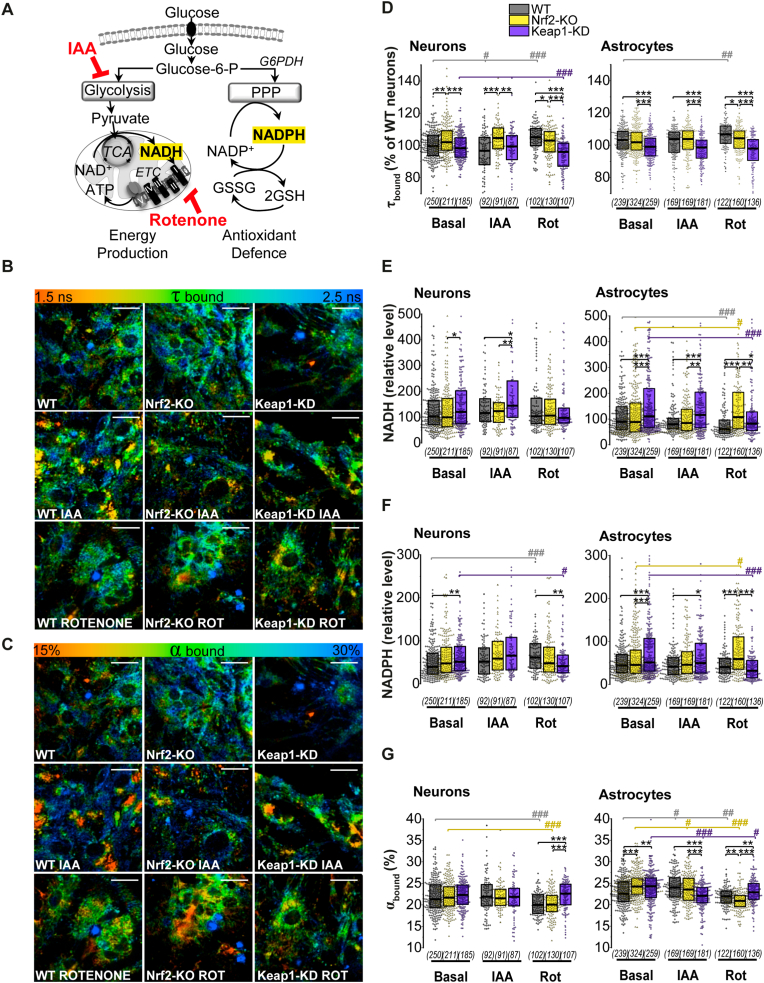
***Inhibition of glycolysis doesn't redirect glucose utilization to pentose phosphate pathway*(A)** Scheme showing main pathways of cellular glucose metabolism and the role of iodoacetic acid (IAA) as an inhibitor of glycolysis (100 μM, 1 h, in the presence of 5 mM sodium pyruvate to maintain viability) and rotenone (rot) (5 μM, 20 min) as an inhibitor of complex I in the electron transport chain (ETC). **(B-C**) Colour-coded representative images of **(D)** NAD(P)H fluorescence lifetime (τ bound) and **(G)** percentage of enzyme-bound NAD(P)H (α bound) in basal conditions and after incubation with IAA or rotenone **(D**–**G)** Quantification in individual neurons and astrocytes of **(D)** NAD(P)H fluorescence lifetime (τ bound); (E) relative cytoplasmic enzyme-bound NADH levels; (F) relative cytoplasmic enzyme-bound NADPH levels and (G) percentage of enzyme-bound NAD(P)H (α bound) in basal conditions and after incubation with IAA or rotenone. Number of cells analysed is shown in brackets. Scale bar: 20 μm. Non-parametric Kruskal-Wallis ANOVA with post-hoc Dunn's test for each group, *p < 0.05, **p < 0.01, ***p < 0.0001. (For interpretation of the references to colour in this figure legend, the reader is referred to the Web version of this article.)

FLIM measurements (Fig. 5) revealed that inhibition of glucose consumption by glycolysis with IAA did not lead however to a higher utilization of glucose in the PPP, as shown by the cytoplasmatic enzyme-bound NADPH levels (Fig. 5 F), which were similar to those in basal conditions. In contrast to the mitochondria, total enzyme-bound cytoplasmatic NADH levels in both neurons and astrocytes mimicked basal conditions despite glycolysis inhibition with IAA, being higher in Keap1-KD cells compared to WT (p = 0.04, p = 10^−4^) and Nrf2-KO (p = 0.006, p = 0.003) (Fig. 5E). Percentage of enzyme-bound NAD(P)H was however reduced in Keap1-KD astrocytes (vs WT p = 10^−4^, vs Nrf2-KO p = 10^−5^) (Fig. 5C, F) suggesting a highly available but less oxidized pool.

On the other hand, inhibition of NADH consumption in mitochondrial respiration with rotenone led to no changes in neurons and a significant decrease of enzyme-bound cytoplasmic NADH levels in WT (p = 0.003) and Keap1-KD astrocytes (p = 10^−5^) (Fig. 5 E). This suggests that production of NADH is reduced in these genotypes when NADH consumption is blocked, probably due to a higher NADH/NAD^+^ ratio. In Keap1-KD neurons and astrocytes, enzyme-bound cytoplasmic NADPH levels were also decreased compared to basal conditions (p = 0.04, p = 10^−9^) when NADH consumption was blocked (Fig. 5F), which could indicate Nrf2 activation induces a reduction in general glucose metabolism when respiration is blocked, rather than diverting glucose for PPP. Importantly, the percentage of cytoplasmic enzyme-bound NAD(P)H (α _bound_) (Fig. 5C, G) diminished in both Nrf2-KO and Keap1-KD astrocytes after inhibition with rotenone and IAA, and probably reflected an increased NADH/NAD ^+^ ratio. α _bound_ was however significantly higher in both rotenone-treated Keap1-KD neurons and astrocytes compared to WT (p = 10^−5^, p = 0.002) and Nrf2-KO (p = 10^−6^, p = 10^−12^), suggesting a higher oxidation of the available pools in cells with constitutive Nrf2 activation when respiration is blocked. In agreement with this, Nrf2-deficient astrocytes showed an opposite profile: inhibition of mitochondrial respiration with rotenone increased both enzyme-bound cytoplasmic NADH (p = 0.01) and NAPDH (p = 0.003) in astrocytes (Fig. 5 E, F), and reduced the percentage of NADPH/NADH bound to enzymes (Fig. 5C, G), suggesting Nrf2-deficiency led to a less oxidized pool.

### 2.5 Nrf2 redirects glucose to NADH production when PPP is inhibited

**Fig. 6:**
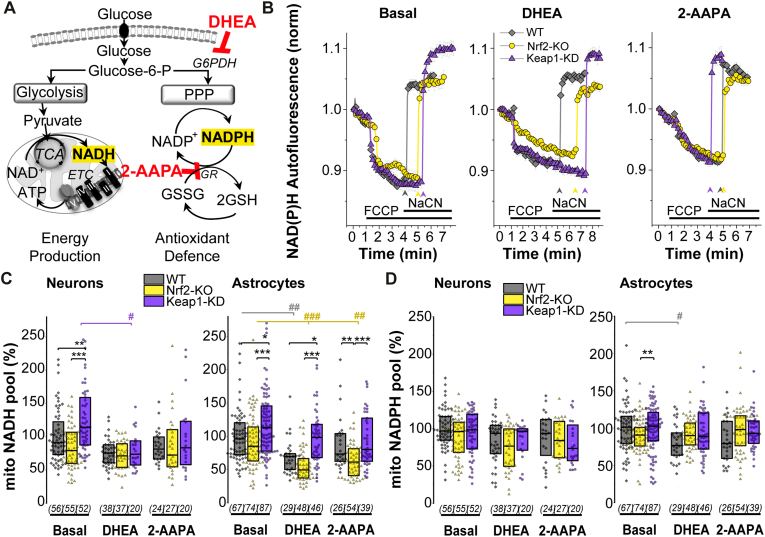
***Mitochondrial NADH and NADPH levels after inhibition of PPP*****(A)** Scheme showing main pathways of cellular glucose metabolism and the role of dehydroepiandrosterone (DHEA) as an inhibitor of pentose phosphate pathway (PPP) (100 μM, 24 h); and 2-AAPA (50 μM, 24 h) as an inhibitor of glutathione reductase (GR) **(B)** Representative traces of mitochondrial NADH homeostasis in astrocytes, in the absence/presence of the inhibitors, with arrows indicating the moment NaCN was added. **(C)** Quantification of the total mitochondrial NADH pool and **(D)** NADPH pool as measured by the remaining NAD(P)H autofluorescence after consumption of all mitochondrial NADH. Number of cells analysed is shown in brackets. Both are expressed as the % of WT basal. Non parametric Kruskal-Wallis ANOVA with post-hoc Dunn's test for each group, *p < 0.05, **p < 0.01, ***p < 0.0001.

**Fig. 7:**
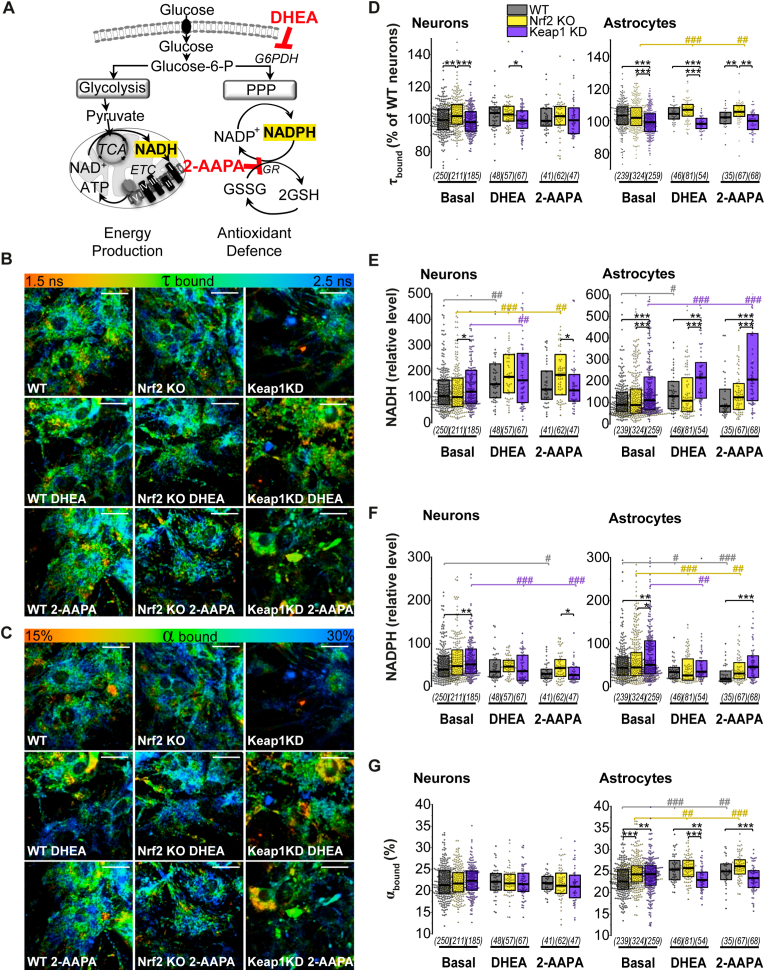
***Nrf2 activation redirects glucose to NADH production when PPP is inhibited* (A)** Scheme showing main pathways of cellular glucose metabolism and the role of dehydroepiandrosterone (DHEA) as an inhibitor of pentose phosphate pathway (PPP) (100 μM, 24 h); and 2-AAPA (50 μM, 24 h) as an inhibitor of glutathione reductase (GR). **(B-C**) Colour-coded representative images of **(D)** NAD(P)H fluorescence lifetime (τ bound) and **(G)** percentage of enzyme-bound NAD(P)H (α bound) in basal conditions and after incubation with DHEA or 2-AAPA **(D**–**G)** Quantification in individual neurons and astrocytes of **(D)** NAD(P)H fluorescence lifetime (τ bound); (E) relative cytoplasmic enzyme-bound NADH levels; (F) relative cytoplasmic enzyme-bound NADPH levels and (G) percentage of enzyme-bound NAD(P)H (α bound) in basal conditions and after incubation with DHEA or 2-AAPA. Number of cells analysed is shown in brackets. Scale bar: 20 μm. Non-parametric Kruskal-Wallis ANOVA with post-hoc Dunn's test for each group, *p < 0.05, **p < 0.01, ***p < 0.0001. (For interpretation of the references to colour in this figure legend, the reader is referred to the Web version of this article.)

We next explored how limiting glucose utilization in the PPP affected its use for energy production in the different genotypes. To this end, we employed dehydroepiandrosterone (DHEA, 100 μM, 24 h) to inhibit glucose-6-phosphate dehydrogenase (G6PDH), the rate-limiting enzyme in PPP (Fig. 6 A). Effect of DHEA in lowering NADPH levels was more profound in astrocytes, where it significantly decreased enzyme-bound cytoplasmic NADPH in all the genotypes (WT p = 0.03, Nrf2-KO p = 10^−4^, Keap1-KD p = 0.009) (Fig. 7 F). This could be explained by a more active PPP in astrocytes as previously observed [8]. In neurons, inhibition of G6PDH and therefore PPP with DHEA only decreased enzyme-bound cytoplasmic NADPH levels in Keap1-KD (p = 10^−4^), making them similar to WT and Nrf2-KO, since they were elevated in Keap1-KD in basal conditions (p = 0.001) (Fig. 7F). This is in agreement with the role of Nrf2 in mediating the transcription of G6PDH in different cell types including neuronal [2,9,25]. In neurons, PPP block led to a higher level of cytoplasmic enzyme-bound NADH in all the genotypes (Fig. 7E), which was however not reflected in the mitochondria, where DHEA decreased mitochondrial NADH in Keap1-KD (Fig. 6 B, C). In astrocytes, PPP inhibition increased cytoplasmic enzyme-bound NADH levels in WT (p = 0.02) and in a significantly higher rate Keap1-KD astrocytes (p = 10^−4^) (Fig. 7 E). Curiously, DHEA resulted in a decrease of mitochondrial NADH in WT (p = 0.005) and Nrf2-KO astrocytes (p = 10^−6^), whereas Keap1-KD cells maintained significantly higher levels (*vs* WT p = 0.01, *vs* Nrf2-KO p = 10^−8^) (Fig. 6 B, C). Under these conditions, WT (p = 10^−5^) and Nrf2-KO (p = 0.002) astrocytes increased the percentage of enzyme-bound cytoplasmic NAD(P)H (Fig. 7 G), suggesting an increased oxidation of the NAD(P)H pool; which remained unchanged in Keap1-KD compared to basal conditions. Taken together, these results suggest Nrf2 redirects glucose for NADH production in astrocytes when PPP is inhibited, highlighting the importance of Nrf2 in providing reducing equivalents for mitochondrial function.

### 2.6 Inhibition of NADPH consumption by glutathione reductase increases NADH in Keap1-KD astrocytes

One of the major consumers of NADPH in brain cells is the enzyme glutathione reductase (GR), which utilizes NADPH to regenerate the oxidized GSH pool (GSSG→GSH) used in antioxidant defence (Fig. 7A). Inhibition of GR in neurons and astrocytes with 2-AAPA (50 μM, 24 h) should therefore result in increased NADPH levels if under normal conditions cells actively consume NADPH via this enzyme. However, in our experiments, incubation of neurons and astrocytes with 2-AAPA did not lead to higher cytoplasmic enzyme-bound NADPH levels, but instead decreased its levels or left them unchanged in the different cell types and genotypes (Figs. 6D and 7F). This might be explained by a potential inhibition of G6PDH induced by an increase in the NADPH/NADP^+^ ratio, leading to a diminished utilization of glucose in the PPP. The possible utilization of NADPH in other reductase systems cannot be discarded either.

In astrocytes, cytoplasmic enzyme-bound NADPH levels were decreased in both WT (p = 10^−6^) and Nrf2-KO (p = 0.003) (Fig. 7 F) when treated with 2-AAPA, while the percentage of enzyme-bound NAD(P)H increased (WT p = 0.01, Nrf2-KO p = 10^−5^), (Fig. 7C, G), suggesting an increased oxidation of the available NADPH/NADH pool. On the other hand, in Keap1-KD astrocytes cytoplasmic enzyme-bound NADPH levels did not change with respect to basal conditions (Fig. 7 F), and glucose was redirected to increase cytoplasmic enzyme-bound NADH pool (p = 10^−5^) (Fig. 7 E). Mitochondrial NADH levels remained significantly higher under conditions of Nrf2 activation compared to deficiency (p = 10^−4^) (Fig. 6C).

Taken together, these results suggest that Nrf2 activation plays an essential role fundamentally in astrocytes but not in neurons, to redistribute glucose utilization from the PPP, when it is either inhibited or down-regulated, to favour the generation of NADH making it available for energy production.

### 2.7 Pharmacological activation of Nrf2 increases NADPH in astrocytes

**Fig. 8:**
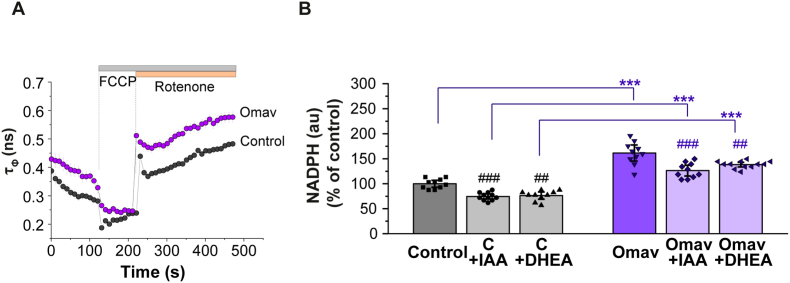
**Pharmacological activation of Nrf2 increases NADPH levels in primary astrocytes.** Primary astrocytes were treated with the pharmacological Nrf2 activator omaveloxolone (30 nM) for 24 h**. (A)** Representative traces showing changes in NAD(P)H fluorescence lifetime in a fast pco.flim FLIM camera (τ Ф) after application of the uncoupler FCCP 1 μM and complex I inhibitor rotenone (5 μM. **(B)** NADPH levels under basal conditions or preincubation with glycolysis inhibitor IAA or PPP inhibitor DHEA. NADPH values were taken as the τ bound level of NAD(P)H fluorescence lifetime after application of FCCP, 1 μM, when all mitochondrial NADH is consumed. Two-way ANOVA with Bonferroni's post-hoc test for each group, ***p < 0.0001 compared to control conditions, ##*p* < 0.01 and ###*p* < 0.001 compared to basal conditions.

In order to investigate if pharmacological activation of Nrf2 leads to the same effects on NADPH levels as the genetic Nrf2 activation (Keap1-KD), we treated primary cortical astrocytes with 30 nM omaveloxolone, for 24 h. Omaveloxolone (Omav) is a potent semi-synthetic triterpenoid Nrf2 activator recently approved by the FDA for treatment of patients with Friedreich's ataxia. In these experiments, we focused on astrocytes based on the findings described above that the effects of genetic Nrf2 activation on the levels of NADH and NADPH were much more pronounced in this cell type. NAD(P)H lifetime measurements were performed using the fast pco.flim FLIM camera that allows measurement of acute FLIM changes in the time of experiment. We hypothesized that changes in τ_Ф_ after application of FCCP, which induces mitochondrial NADH consumption as explained before (Fig. 2), could be representative of the NADPH changes induced by the different conditions, because NADH is minimal under FFCP treatment (Fig. 8A–B). τ_Ф_ values suggested that activation of Nrf2 with Omav also increased enzyme-bound NADPH levels in astrocytes, in a similar way to Keap1-KD (Fig. 8 A-B, Fig. 3E). Preincubation with the PPP inhibitor 100 μM DHEA reduced NADPH levels in both control and omaveloxolone treated astrocytes (Fig. 8B), confirming the previous results in Keap1-KD cells (Fig. 7F). Most importantly, pre-incubation of the cells with the glycolysis inhibitor 20 μM IAA did not increase NADPH in control and Omav-treated astrocytes (Fig. 8B), in agreement with the data obtained with Keap1-KD cells (Fig. 5F), and in this case they were reduced relative to untreated (Fig. 8B). Therefore, pharmacological activation of Nrf2 in astrocytes leads to results similar to those obtained upon genetic upregulation of Nrf2, which further suggests that glucose is not being diverted to support antioxidant defence when glycolysis is inhibited.

## 3 Discussion

Here we show that constitutive Nrf2 activation in Keap1-KD neurons and astrocytes leads to significantly increased glucose uptake. High glucose uptake upon Nrf2 activation has been previously shown for fibroblasts and myocytes [7,26], cells which have different glycolytic activity compared to neurons and astrocytes. Higher glucose uptake in Keap1-KD cells can explain their faster respiratory activity and consumption of NADH and ATP production [12]. Interestingly, Keap1-KD cells had higher glucose uptake independently of the level of glucose in the medium, suggesting higher expression or activity of glucose transporters in these cells, a conclusion further supported by the observed increase in the mRNA levels for GLUT1 in brown adipose tissue of Keap1-KD mice [26].

In basal conditions, Nrf2 activation led to an increase in enzyme-bound NADPH and, more dramatically increased cytoplasmic and mitochondrial NADH levels in neurons and astrocytes. These observations agree with the known roles of Nrf2 activation in antioxidant defence and mitochondrial function [13]. When exposing the cells to lower availability of extracellular glucose, astrocytes appeared to be more sensitive than neurons, and mitochondrial NADH levels were significantly decreased in all the genotypes, especially in the Nrf2-KO astrocytes, although enzyme-bound cytoplasmic NADH didn't change relative to basal conditions. The smaller effect in neurons could be partially explained by glycogen as an energy reservoir in astrocytes that provide active neurons with the glycolytic metabolite lactate [27]. However, when considering the higher glucose uptake in neurons [28], a more likely explanation could be the higher NADH pool in mitochondria of neurons.

Under conditions of low glucose, the levels of cytoplasmic enzyme-bound NADPH decreased in Keap1-KD neurons, leading to similar NADPH levels across all the genotypes in both neurons and astrocytes, suggesting Nrf2 favours the use of glucose for energy production rather than antioxidant defence when glucose availability is low. In contrast, when glucose is not limited and its utilization is stimulated, such as with treatment with orthovanadate, in all the genotypes cytoplasmic enzyme-bound NADH levels remain similar to those in basal conditions, but NADPH levels are enhanced (in both cases Keap1-KD being higher than WT and Nrf2-KO).

Thus, we conclude that Nrf2 activation leads to an increase in glucose uptake in neurons and astrocytes and that glucose consumption is prioritized towards bioenergetic needs when glucose is limited, but metabolized towards PPP/NADPH when glucose stimulation is stimulated (as with OV).This is in contrast with results observed in other cell lines with very different metabolism, such as cancer cells, where Nrf2 redirects glucose to anabolic pathways to support cell proliferation [9].

Importantly, inhibition of glucose utilization in glycolysis didn't lead to a higher utilization of glucose in the pentose phosphate pathway, as shown by the similar cytoplasmic enzyme-bound NADPH levels. However, blocking mitochondrial NADH consumption in the electron transport chain with rotenone, led to a decrease in NADH and a massive increase in mitochondrial NADPH, changes that were independent of Nrf2 in both neurons and astrocytes (Fig. 4). This increase is likely attributable to NADPH production by mitochondrial isocitrate dehydrogenase. Incubation of the cells with rotenone had opposite effects on the level of cytosolic enzyme-bound NADPH – deficiency in Nrf2 led to increase while activation of Nrf2 to a decrease suggesting that Nrf2 blocks the redistribution of glucose metabolism from glycolysis/TCA to PPP in brain cells. On the contrary, when PPP was inhibited, glucose was redirected to cellular NADH production. This was independent of the genotype in neurons, while in astrocytes it was dependent on Nrf2 activity. Importantly, PPP inhibition was also more efficient in astrocytes.

In agreement with previously published results showing that Nrf2 controls the expression of glutathione reductase [[29], [30], [31], [32]], we found that inhibition of this enzyme in Keap1-KD astrocytes led to a sustained higher cytoplasmic enzyme-bound NADPH level, whereas Nrf2 deficiency and WT decreased the level of NADPH. Notably, this effect was not observed in neurons.

Metabolic interactions are a fundamental part of the neuron-astrocytic communication in the brain. Importantly, early in the mouse cortical neuronal development, the expression of *Nfe2l2*, the gene encoding Nrf2, is repressed by epigenetic inactivation of its promoter to allow maturation processes orchestrated by redox-sensitive JNK and WNT signalling pathways [33]. This would highlight the importance of the role of astrocytic Nrf2 in the support of energy and metabolic homeostasis during these stages.

Taken together, these results highlight the important role of Nrf2 in regulating energy metabolism, by prioritizing the distribution of glucose preferentially for energy production in brain cells, when availability is reduced. In addition to physiology, this observation is important for neurodegenerative and other brain disorders where not only oxidative stress, but also energy deprivation is commonly involved in the mechanism of pathology. This role further supports the protective effects of pharmacological Nrf2 activation to prevent neurodegeneration [14,16,17].

## 4 Conclusion

In this study, we found that neurons and astrocytes from Keap1-KD mice have a higher rate of glucose uptake, and levels of enzyme-bound cytoplasmic NADPH and mitochondrial NADH compared to WT or Nrf2-deficient cells. Inhibition of mitochondrial complex I led to a decrease of the mitochondrial NADH pool independently of the genotype, and increased the level of mitochondrial NADPH in neurons and astrocytes. Under these conditions, enzyme-bound cytoplasmic NADPH increased in Nrf2 deficiency (Nrf2-KO) and decreased in Nrf2 activation (Keap1-KD), suggesting Nrf2 might block the redistribution of glucose from glycolysis to PPP when complex I is inhibited. Inhibition of glycolysis decreased mitochondrial NADH, but had no effect on NADPH. Inhibition of the PPP decreased NADPH in astrocytes and decreased mitochondrial NADH in Nrf2-deficient and WT astrocytes, but not in their Keap1-KD counterparts, where the basal level is high. Cytoplasmic enzyme-bound NADH increased in all cell types and genotypes, except Nrf2-KO astrocytes when PPP was inhibited. Inhibition of glutathione reductase decreased cytoplasmic enzyme-bound NAPDH levels, except in Keap1-KD astrocytes, where it additionally increased cytoplasmic enzyme-bound NADH. In contrast to astrocytes, in Nrf2-deficient neurons both cytoplasmic enzyme-bound NADH and NADPH were higher than in Keap1-KD when glutathione reductase was inhibited. Taken together, our results demonstrate that Nrf2 activation enhances glucose uptake in neurons and astrocytes, where glucose consumption is prioritized for mitochondrial NADH and energy production, with smaller contribution to antioxidant pathways. Considering the epigenetic suppression of Nrf2 during neuronal development, this prioritization strategy explains the exquisite vulnerability of neurons to oxidative stress and their reliance on astrocytic Nrf2 for providing reducing equivalents for redox reactions and maintenance of mitochondrial health. Importantly, it can successfully be achieved by pharmacological activation of Nrf2 in astrocytes, which possibly can play a protective role in conditions of ischaemia/reperfusion and energy deprivation.

## 5 Methods

### 5.1 Animals

Wild-type (WT), Nrf2-knockout (Nrf2^−/−^, Nrf2-KO) and Keap1-knockdown (Keap1^flox/flox^, Keap1-KD) mice [34,35] were originally generously provided by Masayuki Yamamoto (Tohoku University, Japan). All mouse colonies were bred and maintained on the C57BL/6 genetic background following ethical approval, in accordance with the regulations described in the UK Animals (Scientific Procedures) Act 1986. The animals were housed at the Medical School Resource Unit of the University of Dundee, with free access to water and food (pelleted RM1 diet from SDS Ltd., Witham, Essex, UK), on a 12-h light/12-h dark cycle, 35% humidity. Of note, the Keap1-KD mice carry two floxed alleles of the *Keap1* gene, which reduces its expression and consequently increases the levels of Nrf2, and thus represent a genetic model for constitutive Nrf2 activation [36,37], and we have previously shown that the enzyme activities of the Nrf2 transcriptional targets NQO1, GST and malic enzyme 1 are upregulated in the cortex of Keap1-KD mice in comparison with their WT counterparts [12].

### 5.2 Cell cultures

**Supplementary Fig. 2:**
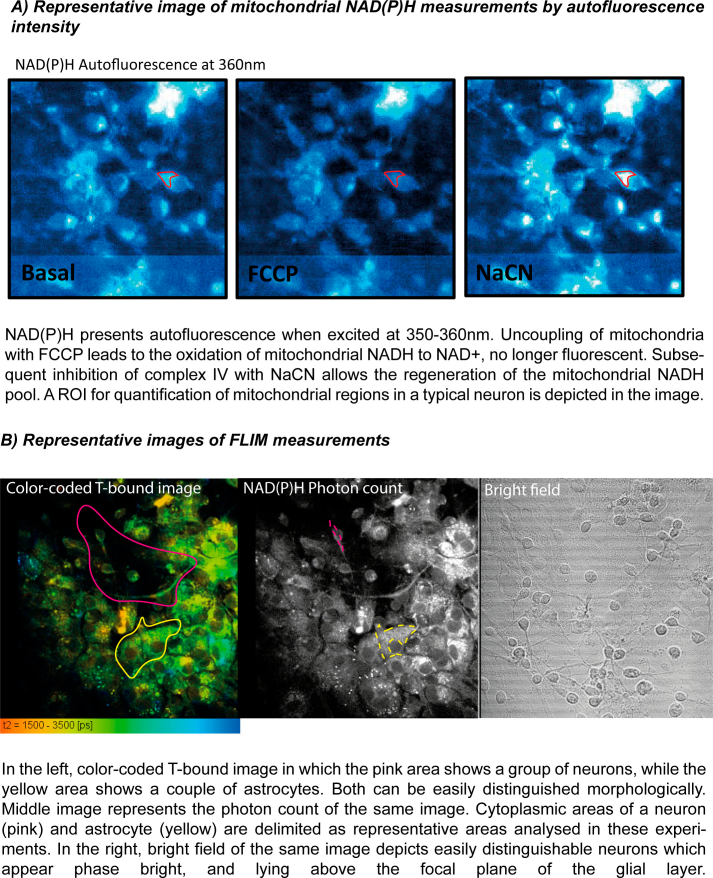
Representative images of NAD(P)H measurements.

Primary cortical mixed cultures of neurons and astrocytes were prepared as described previously [38], with modifications, from the cortex of WT, Nrf2-KO and Keap1-KD mouse pups (P1-P3). Three independent cultures were prepared. Experimental procedures were performed in full compliance with the United Kingdom Animal (Scientific Procedures) Act of 1986 and with the European directive 2010/63/EU. Cortex was dissected and rapidly placed into ice-cold Hank's Balanced Salt Solution (HBSS). The tissue was then trypsinized (0.05% trypsin/EDTA) for 15 min at 37 °C, homogenised, pelleted, resuspended in Neurobasal A media and plated on glass coverslips previously coated with poly-d-lysine (Sigma-Aldrich, Poole, UK). Cultures were maintained at 37 °C in a humidified atmosphere of 5% CO_2_ in Neurobasal A media supplemented with B27 and 2 mM Glutamax and in the presence of penicillin/streptomycin (all reagents from Thermo Fisher Scientific, Paisley, UK). Media was replaced after one week and cells were used at 12–15 DIV in all the experiments. Neurons were easily distinguishable from glial cells in these cultures: they appeared phase-bright, had smooth rounded somata and distinct processes, and typically laid above the focal plane of the glial layer. Representative image shown in Supplementary Fig. 2. Cultures from the three genotypes were similar in terms of cell density, composition and morphology.

For the preparation of astrocytic primary cultures, a similar protocol was used, but cells were plated in T75 flasks and maintained in astroglial media, containing DMEM with 2 mM Glutamax and in the presence of penicillin/streptomycin and 10% fetal bovine serum (FBS), all from ThermoFisher Scientific (Paisley, UK). Cultures were passaged at Day 7 using trypsin and plated in glass coverslips for the experiments.

### 5.3 Glucose uptake

The fluorescent glucose analogue 2-NBDG (Molecular Probes, Thermofisher) was used to monitor glucose uptake in neurons and astrocytes. 2-NBDG is a widely used indicator for glucose uptake, with typical incubation times between 30 min and 1 hour. Once up-taken, some reports show that 2-NBDG is phosphorylated and decomposed to a non-fluorescent derivative, so it is assumed the fluorescence intensity reflects a dynamic equilibrium between uptake and decomposition. Images were acquired using a Zeiss 710 VIS CLMS confocal microscope equipped with a META detection system and an ×40 oil immersion objective (Zeiss, OberKOchen, Germany). The 488 nm laser was used to excite 2-NBDG and emitted fluorescence was measured above 495 nm. Two parameters were estimated: the rate of fluorescent glucose uptake by cells over time (shown in Fig. 1) and the total fluorescent glucose uptaken by cells after 30 min (shown in Suppl. Fig. 1). Unless otherwise stated, immediately before the start of the experiment, media was replaced with HBSS containing low glucose (1 mM), and a couple of images were taken to estimate basal green autofluorescence. 200 μM 2-NBDG was then applied, leading to an increase in green fluorescence in the extracellular media. To estimate the rate of uptake of fluorescent glucose inside neurons and astrocytes, the increase in green fluorescence inside individual cells over time was quantified using Zeiss software. Results were corrected by the initial green autofluorescence prior to 2-NBDG addition, and the rate of glucose uptake (au/min) was normalized to WT neurons in normal conditions (taken as 100%), and expressed in percentage.

Additionally, in some experiments (Suppl Fig. 1), extracellular 2-NBDG was washed after 30 min incubation and Z-stacks were taken to measure green fluorescence intensity and quantify the total amount of up-taken dye inside the cells. Results were corrected by the initial green autofluorescence prior to 2-NBDG addition and normalized to WT neurons with normal glucose, and expressed in percentage.

### 5.4 Mitochondrial NADH homeostasis

NAD(P)H autofluorescence was measured as previously described [21] using an epifluorescence-inverted microscope equipped with a ×40 oil objective (Nikon Eclipse Ti-S). A Xenon arc lamp passed through a monochromator was used to provide excitation light at 360 nm. Emitted light was reflected through a 455 nm long-pass filter to an Andor Zyla sCMOS camera (Cairn Research, Kent, UK). Images were acquired and analysed using Andor Software. After recording basal autofluorescence, 1 μM FCCP was added to completely depolarize the mitochondria and oxidize the mitochondrial pool of NADH to NAD+, which is no longer fluorescent. This point was taken as 0% mitochondrial NADH and remaining fluorescence at this stage was taken as coming from mitochondrial NADPH. 1 mM NaCN was then added to inhibit respiration and allow the regeneration of the mitochondrial pool of NADH (100%). Mitochondrial NADH pool was calculated as the difference between maximum and minimum values of autofluorescence in the mitochondria. NADH redox index was calculated as the % represented by the basal levels when extrapolating its value in the 0–100% range generated by FCCP and NaCN respectively. Calculations of mitochondrial NADH and NADPH pools was performed on the raw data of autofluorescence intensity, after subtracting the background. Values were then normalized to the controls for each set of experiments, so controls are 100%. Regions of interest analysed in these experiments in the individual cells correspond only to mitochondria, which is clearly distinguished by the changes in NADH levels induced by the mitochondrial uncoupler and inhibitor FCCP and NaCN, although a minimal implication of cytosolic NAD(P)H cannot be excluded. Representative images of the experiment are shown in Supplementary Fig. 2.

### 5.5 FLIM measurements

Two different microscopy set-ups were employed for all the FLIM experiments performed in the primary neuronal-glia co-cultures from WT, Nrf2-KO and Keap1-KD animals. The same experimental and acquisition conditions were maintained across the experiments no matter the microscopy set-up used and results were normalized when appropriate to WT neurons. Time-domain NAD(P)H FLIM was performed using either an upright LSM510 microscope (Carl Zeiss) equipped with a 40x (NA 1.0) water-dipping objective, or an inverted Zeiss LSM710 microscope equipped with a 40x oil immersion objective. In both cases, samples were excited with a Ti:sapphire laser (Chameleon Ultra, Coherent) tuned to 720 nm. Fluorescence was detected by a hybrid photomultiplier tube (HPM-100, Becker & Hickl) after passing through a 435–485 nm emission filter. Emission events were registered by a time-correlated single photon counting module (SPC-830 for the LSM510 and SPC-150 for the LSM710, Becker & Hickl) while scanning continuously for 2 min with a pixel dwell time of 1.6 μs, resulting in 256 x 256 pixel FLIM images containing between 101 and 103 photons per pixel. 5 × 5 binning was applied to increase signal to noise before least squares fitting of a biexponential decay function in SPCImage 3.0.8 (Becker & Hickl) by iterative reconvolution with an instrument response function (IRF) recorded from the 460 nm s harmonic generation signal of a potassium dihydrogen phosphate (KDP) crystal at 920 nm excitation. A constant (time-uncorrelated) offset was allowed to float at each pixel to account for background fluorescence and the IRF was translated across the time axis to compensate for its dependence on excitation wavelength. Mean parameter values across cellular regions of interest were measured by exporting the data to ImageJ (NIH). Regions of interest correspond to the cytoplasm, nucleus was easily distinguished and excluded from the measurements. Representative images of the experiment are shown in Supplementary Fig. 2.

### 5.6 FLIM measurements in primary astrocytes treated with the pharmacological Nrf2 activator

The fluorescence lifetime parameters in the primary culture of astrocytes were measured by pco.flim FLIM camera (PCO AG, Germany). In the CMOS full-frame camera with a resolution of 1008 x 1008 pixels, the measurements are implemented in the frequency domain. For imaging, the NAD(P)H fluorescence in cells, UV laser (375 nm/10 mW) with the amplitude modulation at the frequency of 40 MHz was used. A liquid light guide (PCO AG, Germany) was used to deliver the radiation from the laser to the Olympus IX73P1F microscope (Olympus Corp., Japan) and mitigate the impact of the speckles on the quality of the image. Images of cells were obtained and processed with the NIS Elements AR version 5.11 software with the pco.flim plugin. The imaging was conducted with the fluorescence filters DC/ET455/50 m and Optoscan P130/ELE/450 dichroic (Cairn Research Ltd., UK) before the camera detector. The obtained images of the fluorescence intensity and the fluorescence lifetimes were processed in ImageJ at the final stage. Statistical analysis was performed using Origin 2019 software (Microcal Software Inc., Northampton, MA, USA).

### 5.7 Statistical analysis

OriginPro 2019 was used for graphs generation and statistical analysis. Data sets were first probed for normality with the Shapiro-Wilk test and homogeneity of variances was analysed with Levene's test. In most of the cases, the data sets did not show a normal distribution, therefore, non-parametric tests were performed (Kruskal-Wallis H test). When appropriate, ANOVA followed by Bonferroni post-hoc test was performed to estimate the statistical significance between experimental groups. A p value less than 0.05 was considered as statistically significant for either test. Histograms represent the mean and error bars the 95% confidence intervals, while box plots represent the median and 25 and 75 percentiles, with the distribution profiles showing single-cell values, unless otherwise indicated. Representative traces are illustrated as the mean of the values of all the individual cells measured in one independent experiment, with error bars indicating the SEM.

## Author contributions

NE, OS, YZ, and TB performed research. NE, OS, EZ, TB and AYA analysed the data. NE, AYA, ATDK, TB, CW and MD wrote the manuscript. NE, ATDK, TB and AYA designed the research.

## Declaration of competing interest

A.T.D.K. is a member of the scientific advisory board of Evgen Pharma. C.W. is employee of Reata Pharmaceuticals.

## Data Availability

Data will be made available on request.
